# Amine-Containing Membranes with Functionalized Multi-Walled Carbon Nanotubes for CO_2_/H_2_ Separation

**DOI:** 10.3390/membranes10110333

**Published:** 2020-11-10

**Authors:** Yutong Yang, Yang Han, Ruizhi Pang, W.S. Winston Ho

**Affiliations:** 1 William G. Lowrie Department of Chemical and Biomolecular Engineering, The Ohio State University, 151 West Woodruff Avenue, Columbus, OH 43210-1350, USA; yang.4249@buckeyemail.osu.edu (Y.Y.); han.779@osu.edu (Y.H.); pang.123@osu.edu (R.P.); 2 Department of Materials Science and Engineering, The Ohio State University, 2041 College Road, Columbus, OH 43210-1350, USA

**Keywords:** carbon dioxide separation, mixed-matrix membrane, amino-functionalized multi-walled carbon nanotube, membrane compaction

## Abstract

Amine-containing mixed-matrix membranes incorporated with amino-functionalized multi-walled carbon nanotubes (AF-MWNTs) were synthesized for CO_2_/H_2_ separation based on the facilitated transport mechanism. AF-MWNTs were chosen primarily as the mechanical reinforcing filler to enhance the membrane stability. At 107 °C and 0.2-MPa feed pressure, the membrane incorporated with 10 wt.% AF-MWNTs showed a CO_2_ permeability of 3196 Barrers and a CO_2_/H_2_ selectivity of 205. At the higher feed pressure of 1.5 MPa, owing to the carrier saturation phenomenon, the same membrane exhibited reduced transport performance with a CO_2_ permeability of 776 Barrers and a CO_2_/H_2_ selectivity of 31. These separation performances at both the low and high feed pressures were well above the theoretical upper bound. Furthermore, the incorporation of 10 wt.% AF-MWNTs led to a significant improvement on membrane stability. The transport performance and selective layer thickness of this membrane maintained for 100 h, which suggested that the incorporation of AF-MWNTs improved the resistance to membrane compaction upon a high feed pressure. Therefore, this work is considered as one of the crucial steps to enable the application of facilitated transport membranes to high-pressure gas processing such as syngas purification.

## 1. Introduction

Hydrogen has attracted great attention in recent years, not only because it is widely considered as a promising clean energy resource, but also because there is an enormous demand for the energy and chemical industries [1]. Currently, most of the hydrogen in the world is produced by hydrocarbon steam reforming, followed by the water-gas shift (WGS) reaction, where CO is converted to additional H_2_ and CO_2_ at a high pressure of 1.5 MPa or greater [2,3,4]. In order to enhance the conversion of CO and obtain high-purity H_2_ stream, it is necessary to develop an effective technology to separate CO_2_ from H_2_ at high pressure.

Compared to other technologies for CO_2_/H_2_ separation, such as amine scrubbing, pressure, or temperature swing adsorption, and cryogenic fractionation, membrane-based technology has advantages in energy efficiency, ease of operation, and low capital cost in addition to its ability to overcome thermodynamic equilibrium limitations [5,6]. Among the advanced polymeric membranes, facilitated transport membranes (FTMs) usually possess both high CO_2_ permeability and high CO_2_/H_2_ selectivity, owing to their possession of reaction to enhance mass transfer. The transport of CO_2_ is mainly facilitated by the reversible reactions between carriers and CO_2_ molecules, while H_2_ follows only the solution-diffusion mechanism with a much lower flux. CO_2_ can also follow the solution-diffusion mechanism; however, the flux by the solution-diffusion mechanism is much lower than that by the facilitated transport mechanism [7,8].

In order to achieve the feasibility in industrial gas processing, the mechanical, thermal, and chemical stabilities of FTMs should be taken into consideration. Several researchers have demonstrated the long-term stability of FTMs under a feed pressure of 0.2 MPa with different carriers [8,9,10]. However, it is still a tremendous challenge to meet the industrial demands at a high pressure of 1.5 MPa or greater and temperatures within the range of 100 to 200 °C. As one of the most commonly employed polymer for the synthesis of FTMs, polyvinyl alcohol (PVA) is typically crosslinked to serve as the polymer matrix not only because of its high hydrophilicity but also excellent compatibility with most CO_2_ carriers and good film-forming ability [11,12]. Unfortunately, the glass transition temperature (Tg) of crosslinked PVA (85 °C) is much lower than the membrane operating temperature, resulting in the susceptibility of the membrane to compaction upon a feed pressure over 1.5 MPa [8,13]. As the membrane is compressed, the free volume of polymer decreases, which causes reductions of CO_2_ permeability and CO_2_/H_2_ selectivity. As reported by Zhao et al., a PVA-based FTM exhibited a 50% reduction in the membrane thickness over a course of 500 h at 106 °C and 1.5-MPa feed pressure, which was accompanied by a severe reduction in both of the CO_2_ permeability and CO_2_/H_2_ selectivity [8].

In order to resist the membrane compaction and maintain the gas separation performance under a high feed pressure, a type of improved FTM has been developed by incorporating mechanical reinforcement nanofillers. Multi-walled carbon nanotubes (MWNTs) have been considered as one of the ideal nanofillers to reinforce the overall membrane mechanical strength [14,15]. Deng et al. demonstrated that incorporating 2 wt.% MWNTs in a polyvinylamine (PVAm)/PVA blend could reduce the compaction of the membrane at elevated pressures and high swelling degrees [16]. Zhao et al. elucidated that acid treatment of MWNT could improve its compatibility with PVA matrix, which enhanced the stability and transport performance of the membranes [14]. To further enhance its compatibility, the hydrophobic graphene walls and tube ends of MWNTs were decorated with amino groups, i.e., amino-functionalized MWNTs (AF-MWNTs), by Ansaloni et al. [17]. AF-MWNT mixed matrix membranes containing PVAm (fixed carrier), AIBA-K, and KOH (mobile carriers) exhibited great transport performance.

The present paper is concerned with an extension to Ansaloni et al.’s work, by investigating the AF-MWNT loading effect on performances of amine-containing membranes. To further enhance the stability and transport performance of the membrane, it is worthwhile to incorporate a higher loading of AF-MWNTs. By incorporating various amounts of AF-MWNTs, the optimal membrane composition would be obtained, which could possess excellent transport performance along with sufficient resistance to membrane compaction upon high pressure.

## 2. Experimental

### 2.1. Materials

Pristine MWNTs were purchased from Cheap Tubes, Inc. (Grafton, VT, USA); the MWNTs were 10–50 μm in length, 8–15 nm in diameter and had >95% purity. PVA (Poval S-2217, 92% purity, MW 150,000 Da, 87–89% hydrolyzed) was provided by Kuraray America, Inc. (Houston, TX, USA); it was a copolymer of vinyl alcohol (98 mol%) and a sulfonic acid sodium salt (2 mol%) [18]. Potassium hydroxide (KOH, pellets, 90%) and potassium bromide (99+% metals basis, FTIR grade) were purchased from Sigma-Aldrich (Milwaukee, WI, USA). Glutaraldehyde (50% solution) was bought from VWR International (Radnor, PA, USA). The 2-Aminoisobutyric acid (AIBA, 98%) was obtained from Alfa Aesar (Ward Hill, MA, USA). The 3-Aminopropyltriethoxysilane (APTES, 99%) was acquired from Thermo Scientific (Madison, WI, USA). Hydrochloric acid (36.5%), extra pure sulfuric acid (98%), trace metal grade nitric acid (69.4%), and acetone (99.7%) were obtained from Fisher Scientific, Inc. (Pittsburgh, PA, USA). Lupamin^®^ 9095 was donated by BASF (Vandalia, IL, USA); it was a commercial liquid product containing 7 wt.% PVAm and 14 wt.% sodium formate [19]. All chemicals were used as received without further purification except AIBA. The primary amino group of AIBA was deprotonated by mixing with a stoichiometric amount of KOH for 24 h before usage [8]. 

A nanoporous polysulfone (PSF) support (average pore size of 9 nm, porosity of about 7%, and thickness of 140 μm including non-woven fabric) was purchased from Microdyn-Nadir US Inc. (formerly TriSep Corporation, Goleta, CA, USA). Hydrophobic microporous polytetrafluoroethylene (PTFE) membrane (pore size ≥ 200 nm with a thickness of 30 μm) was bought from Sumitomo Electric Interconnect Products, Inc. (San Marcos, CA, USA).

All certified-grade gas cylinders were purchased from Praxair Inc. (Danbury, CT, USA). A gas mixture (20% CO_2_, 40% H_2_ and 40% N_2_) was used as feed gas for the transport measurement. Pre-purified argon was used as the sweep gas for the transport measurement and the reference gas for gas chromatography.

### 2.2. Dispersion and Modification of MWNTs

**Figure 1:**
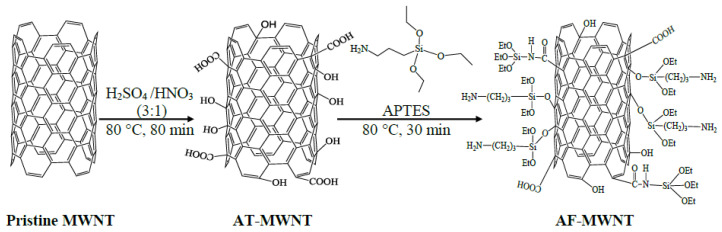
Scheme of the procedure for multi-walled carbon nanotubes (MWNT) modification.

The procedure of MWNT modification is shown in Figure 1. First, 0.5 g of pristine MWNTs was immersed in a 120-mL mixture of HNO_3_/H_2_SO_4_ (3:1, *v/v*) to form an aggressively oxidizing environment [20]. The suspension was ultrasonicated for 3 h (FS-30, 130 W, Fisher Scientific), then refluxed at 80 °C for 80 min. The suspension was then continuously washed with reverse osmosis (RO) water against the PTFE membrane under vacuum until the pH of the filtrate reached 5 to remove the remaining acids. The product was dried overnight and then dispersed in RO water—(10 mg/mL) by an ultrasonic bath for 2 h to disentangle the aggregation. After the oxidation procedure, the resultant –COOH and –OH groups on the surfaces of the sidewalls and tube ends of acid-treated MWNTs (AT-MWNTs) were further reacted with APTES, an aminosilane, to afford the AF-MWNTs [17,21]. In the reaction, acetone was used as the solvent to dilute APTES (5 wt.%) to prevent the self-condensation of APTES. The solution was mixed with the AT-MWNT dispersion at a weight ratio of 1:10 (APTES/AT-MWNT) and then refluxed at 80 °C for 30 min. The mixture was further ultrasonicated for 2 h and then centrifuged at 6000 rpm for 10 min to remove any aggregates. The final AF-MWNT concentration was measured by a drying method. 

### 2.3. Membrane Synthesis

In the present work, mixed-matrix membranes were synthesized by a solution coating method [8,22]. Firstly, crosslinked PVA-POS was synthesized by a sol-gel reaction between PVA and APTES, followed by the KOH-catalyzed crosslinking reaction with glutaraldehyde, as described in our previous paper [22]. The scheme of the reaction pathway has also been shown in our previous publications [8,22]. Secondly, the prepared AF-MWNT dispersion was added dropwise to the crosslinked PVA-POS solution, aiming for 0–10 wt.% AF-MWNTs loading in the final total solid of the coating solution. Then, the mixture underwent ultrasonication for 3 h to obtain a well-dispersed solution. The corresponding amounts of KOH solution, AIBA-K solution, and Lupamin^®^ 9095 were added to the dispersion in sequences to obtain a homogenous coating solution without gelling. The solid composition of the final coating solution, excluding the AF-MWNTs, was 20.6 wt.% PVA, 5.5 wt.% APTES, 19.4 wt.% KOH, 31.7 wt.% AIBA-K, and 22.8 wt.% Lupamin^®^ 9095. When the loading of AF-MWNTs was included and increased, the solid concentrations of other components were proportionally decreased. 

AIBA-K and KOH served as mobile CO_2_ carriers in the membrane, the PVAm in Lupamin^®^ 9095 was used as the fixed-site carrier, and AF-MWNTs were employed as reinforcing nanofillers. The final solution was centrifuged at 8000 rpm for 10 min to remove aggregates and gas bubbles. Finally, the coating solution was coated on a flat-sheet PSF support using a Gardco adjustable micrometer film applicator (Paul N. Gardner Company, Pompano Beach, FL, USA) to obtain a selective layer thickness of 17 μm after drying. After coating, the membrane was immediately placed in a fume hood, followed by curing in a Thermolyne 30,400 muffle furnace (Thermo Scientific, Waltham, MA, USA) at 120 °C for 6 h to complete the crosslinking reaction. The thicknesses of the membranes were measured by an electronic indicator (Model 543-252B, Mitutoyo America Corporation, Aurora, IL, USA) with an accuracy of ±0.5 μm. 

### 2.4. Characterization

Fourier transform infrared (FTIR) spectra of pristine MWNTs, AT-MWNTs, and AF-MWNTs were recorded using a Nicolet iS50 FTIR spectrometer (Thermo Fisher Scientific Co., Waltham, MA, USA). To prepare the FTIR samples, finely ground nanotubes were dispersed in KBr and pressed into pellets.

The functional groups on the surfaces of MWNTs were characterized by X-ray photoelectron spectroscopy (XPS). The spectra were collected by a Kratos AXIS Ultra photoelectron spectrometer, which was equipped with a monochromatic (Al) X-ray gun operated at 15 keV and 120 W. The pass energy was fixed at 80 eV for the general element survey, while a value of 20 eV was used for high-resolution core-level spectra. The C 1s and O 1s regions were swept for four times, while the N 1s and Si 2p regions were scanned for 16 times due to their sparsity. All the spectra were calibrated with a charge reference to adventitious C 1s peak at 284.5 eV.

The cross-section morphologies of the composite membrane before and after the gas permeation test were characterized using the FEI Apreo LoVac high resolution scanning electron microscope (SEM) (Thermo Fisher Scientific Co., Waltham, MA, USA). The sample preparation was done under cryogenic conditions using liquid nitrogen.

The gas permeation performance was characterized as described in our previous publications [8,14]. In brief, the membrane was placed into a rectangular stainless-steel permeation cell with an effective area of 2.7 cm^2^. A counter-current feed and sweep flow configuration were applied to increase the driving force. The gas flow rates of the feed and sweep were controlled by mass flow controllers. In our previous work, an optimal operating temperature of 107 °C was found for amine-containing membranes operated under 1.5 MPa [17,22]. Therefore, the testing was carried out at 107 °C inside a temperature-controlled oven. Two water pumps were used to keep the levels of relative humidity (RH) for two sides of the cell, respectively. The operation conditions are listed in Table 1.

After the feed and sweep streams reached steady state, the retentate and permeate streams were dried and then sent to an Agilent 6890N gas chromatograph (Agilent Tech., Palo Alto, CA, USA) for the measurements of gas compositions. The gas permeation performance was expressed in terms of CO_2_ permeability and idea CO_2_/H_2_ selectivity. The selective layer thickness was 17 µm for all the membranes, and the mass transfer resistance due to the porous polymer support was negligible. Therefore, the CO_2_ permeability was calculated by multiplying the CO_2_ permeance of the composite membrane with the selective layer thickness. The permeability values were reported with a unit of Barrer (1 Barrer = 1 × 10^–10^ cm^3^(STP) cm cm^–2^ s^–1^ cmHg^–1^) [8,14,15,23].

## 3. Results and Discussion

### 3.1. Characterization of MWNTs

**Figure 2:**
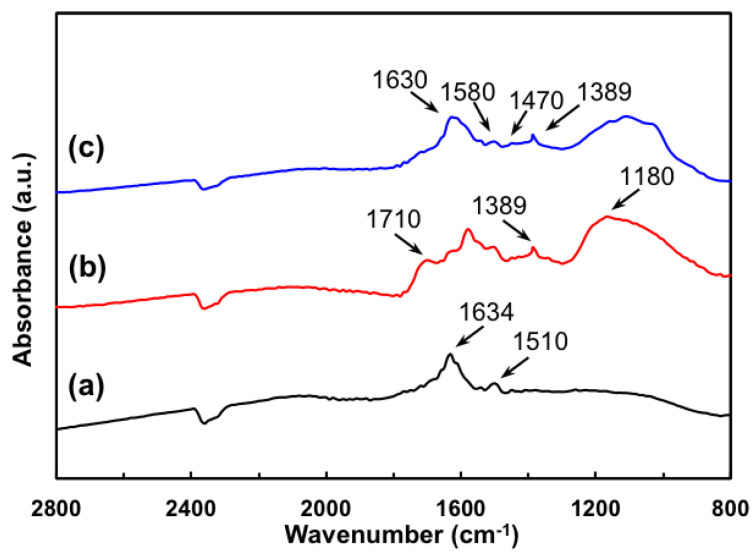
FTIR spectra of (**a**) pristine MWNTs, (**b**) acid-treated multi-walled carbon nanotubes (AT-MWNTs), and (**c**) amino-functionalized multi-walled carbon nanotubes (AF-MWNTs).

Figure 2 illustrates the FTIR spectra in the range of 800–3000 cm^–1^ of pristine MWNTs, AT-MWNTs, and AF-MWNTs. For the pristine MWNT sample, the band signal at 1634 cm^–1^ is assigned to the C=O stretching mode of quinone groups, while the C=C bond signal at 1510 cm^–1^ is attributed to the graphitic carbon skeleton [24,25].

The oxidation process created some defects at the sidewalls and open ends of the MWNTs, which were attached with oxygenated functional groups. As shown in Figure 2b, the two new peaks appearing at 1710 cm^–1^ and 1180 cm^–1^ are assigned to the C=O and C–O stretching vibrations of the carboxylic groups (–COOH), respectively [26,27,28]. The peak at 1389 cm^–1^ is attributed to the O–H stretching in the –COOH and –OH groups [26]. These features confirmed the attachment of –COOH and –OH groups on the MWNTs after the acid treatment. Figure 2c shows the spectrum of the AF-MWNTs. The disappearance of the band at 1710 cm^–1^ was due to the amide carbonyl stretching vibration mode, and the band at 1630 cm^–1^ was resulted from two overlapped bands, which are attributed to the C=O stretching mode of the quinone groups and that of the amide groups. The peak at 1389 cm^–1^ represented the unreacted, remaining –COOH and –OH groups on AF-MWNTs. The new band at 1580 cm^–1^ corresponded to –NH_2_ scissors vibrations, and the weak peak at 1470 cm^–1^ was from –NH bending vibrations of amide groups [29]. The broad bands in the range of 1000–1200 cm^–1^ are assigned to the Si–O–Si and Si–O–C_x_H_y_ bridges [22,29]. The appearance of these signals confirmed the condensation reaction of carboxylic acid groups/hydroxyl groups with silanol groups. Therefore, the AF-MWNTs were successfully synthesized. It should be noted that the spectra beyond 2800^–1^ cm are not shown in Figure 2. The hydroxyl groups (–OH) on the surface of the MWNTs, which resulted from either the oxidation during the purification of the raw material or the atmospheric moisture, rendered broad peaks at 3000–3500 cm^–1^ and masked any weak peaks from the N–H stretching of the amino groups. 

**Figure 3:**
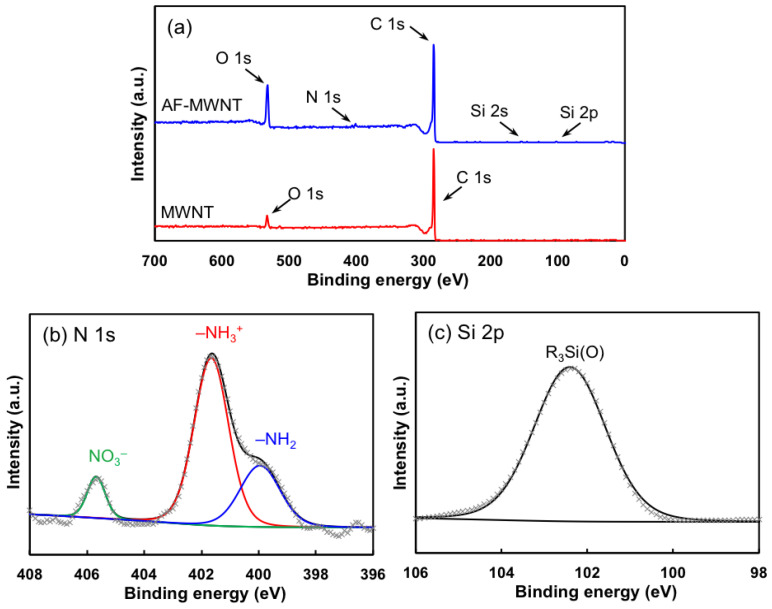
(**a**) X-ray photoelectron spectroscopy (XPS) survey spectra of MWNT and AF-MWNT. The spectrum of AF-MWNT is offset by 50%. (**b**) Spectral decomposition of the N 1s region of AF-MWNT. (**c**) Spectral decomposition of the Si 2p region of AF-MWNT. The black solid lines in (**b**,**c**) are the fitted envelopes and the gray markers are the spectroscopic data.

The XPS spectroscopic data of the pristine MWNT and AF-MWNT are shown in Figure 3a. The spectrum of AF-MWNT is offset by 50% for the ease of comparison. The pristine MWNTs only exhibited features in the C 1s and N 1s regions. An elemental analysis revealed that the sample surface consisted of 96.09% carbon atoms and 3.91% oxygen atoms; the presence of oxygen was likely due to adventitious contamination or water [30]. The acid treatment and amino-functionalization enhanced the oxygen fraction to 13.03% with a reduced carbon content of 85.61%. Moreover, nitrogen and silicon atoms were observed, and they account for 0.66% and 0.70% of the surface atomic composition, respectively, which were slightly lower than the (N):(Si) ratio in the amine coupling agent APTES. 

The deconvolution of the N 1s peak of AF-MWNT in Figure 3b shows a primary peak at 401.6 eV, accompanied by a secondary peak at a lower binding energy of 399.9 eV. These two peaks are attributed to the protonated (–NH_3_^+^) and unprotonated (–NH_2_) amines, respectively [31]. The high protonation degree of the amino groups (ca. 70%) is expected due to the acidic carboxyl groups created by the acid treatment. An additional peak at 405.7 eV is assigned to certain nitrate residual [32], which is originated from the nitric acid used during the acid treatment. 

Figure 3c shows the Si 2p core-level spectrum of the AF-MWNTs sample. Due to the absence of elemental silicon Si^0^ (binding energy ca. 99 eV), the spectrum was fitted with a singlet instead of using Si 2p_1/2_ and Si 2p_3/2_ doublet [33]. As seen, only one peak is determined at 102.4 eV, which is assigned to the silane bound (R_3_Si(O)) formed between APTES and the acid-treated MWNT [34,35]. Both the FTIR and XPS results verify the presence of the amino groups on the surface of MWNTs.

### 3.2. Effect of AF-MWNT Loading on the Amine Content of the Membrane

Based on the XPS spectroscopic data, nitrogen atoms were observed and accounted for 0.66% of the surface atomic composition for AF-MWNTs. According to the estimated number of rolled layers of graphene of the MWNTs, the amine concentration value for the AF-MWNT was approximately 0.09 mmol/g, which was sufficiently low and could be neglected in the total membrane amine concentration value [36].

Table 2 lists the calculated amine concentration (AC) values for amine carriers, which were calculated based on the molecular structures. For example, PVAm has an AC value of 23.3 mmol/g. However, it is worth noting that for the mobile carrier AIBA-K, its amine concentration needs to be doubled because AIBA-K is a sterically hindered amine, which reacts with CO_2_ with a 1:1 stoichiometric ratio instead of the 2:1 ratio for an unhindered amine [22]. The AC value of APTES was calculated based on the structure of SiO_1.5_(CH_2_)_3_NH_2_, assuming that the aminosilane was completely converted into aminosilica via the sol-gel process after the 6-h curing [22]. 

**Figure 4:**
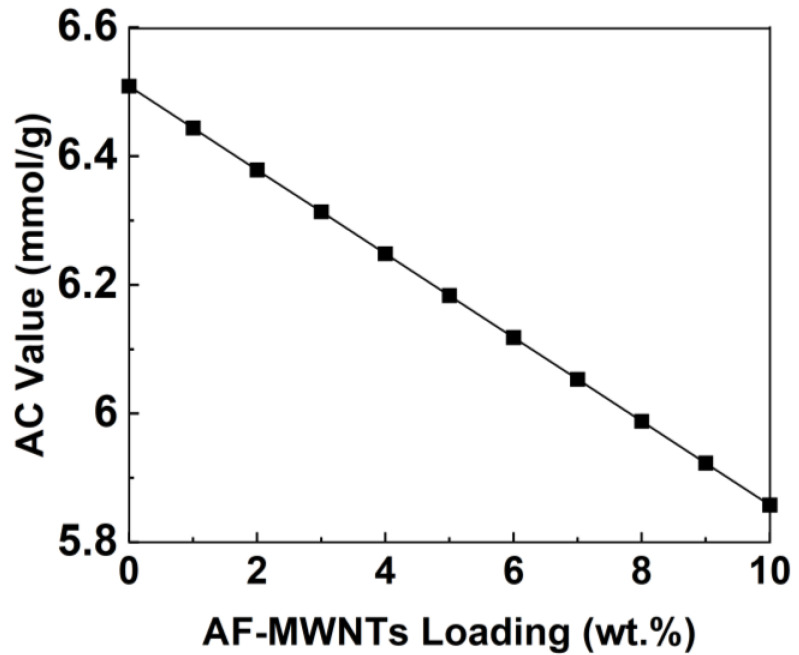
Calculated AC values of the membrane at different AF-MWNT loadings.

Figure 4 illustrates the effect of AF-MWNT loading on the AC value in the membrane. The membrane AC value followed a quantifiable linear decrease as the concentration of AF-MWNT increased. If other factors are not taken into consideration, the decrease of the AC value will lead to the decrease of both CO_2_ permeability and CO_2_/H_2_ selectivity, which can be explained by the fact that the amount of amines available for the facilitated transport of CO_2_ is reduced. This dependency of the AC value could be helpful to explain the effect of AF-MWNTs on the separation performance, which will be discussed in the following section. 

### 3.3. Effect of AF-MWNT Loading on Membrane Performance at a Low Feed Pressure

**Figure 5:**
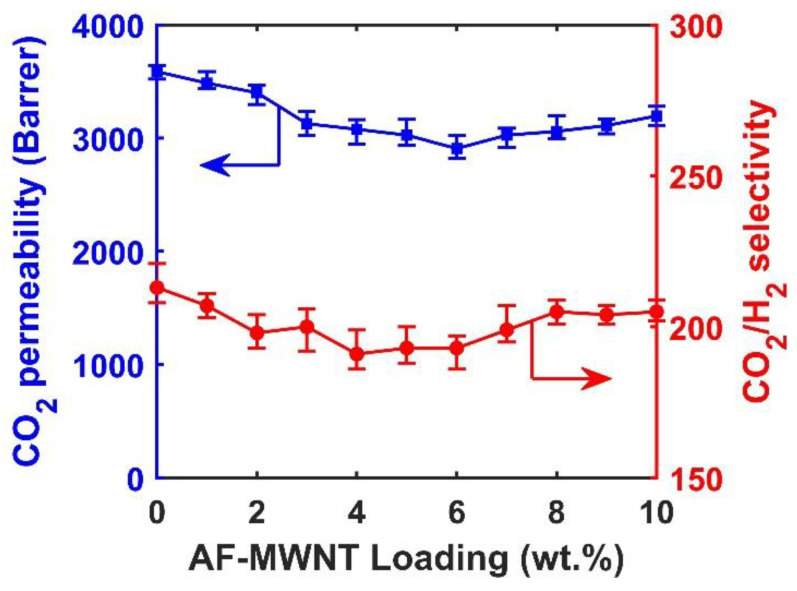
Effects of AF-MWNT loading on CO_2_ permeability and CO_2_/H_2_ selectivity under 0.2-MPa feed pressure: CO_2_ permeability (■) and CO_2_/H_2_ selectivity (●). Error bars represent the minimum and maximum values of three samples.

The effect of AF-MWNT loading on the gas transport performance of the FTM was investigated under two different operating pressures. At a low feed pressure of 0.2 MPa, the CO_2_/H_2_ selectivities and CO_2_ permeabilities of membranes with different AF-MWNT loadings at 107 °C are shown in Figure 5. The CO_2_/H_2_ selectivity and CO_2_ permeability continuously reduced when the concentration of AF-MWNT increased from 0 to 6 wt.%. At 6 wt.% AF-MWNTs, the membrane exhibited a CO_2_/H_2_ selectivity of 193 and a CO_2_ permeability of 2907 Barrers. The incorporation of AT-MWNTs should always result in reduced CO_2_ permeability due to the decreased AC value. 

However, as the concentration of AF-MWNT further increased beyond 6 wt.% and to 10 wt.%, the CO_2_ permeability started to increase and finally reached 3196 Barrers, which was similar to the results of the membrane with 3 wt.% AF-MWNTs, but still less than the results of the membrane without nanofillers. The increased CO_2_ permeability with the increased nanofiller loading beyond 6% can be explained by the following two reasons. Firstly, the size of AF-MWNTs was small enough to insert into the spacings of polymer chains, which increased the free volume and disrupted the polymer-chain packing thus enhanced gas diffusion [37]. Secondly, according to the atomistic simulation results, the CO_2_ transport diffusivity inside AF-MWNTs is extremely rapid, which means the tubular AF-MWNT itself can serve as gas diffusion tunnels [38]. However, if more AF-MWNTs were incorporated into the membrane, the CO_2_/H_2_ selectivity exhibited a precipitous decline to only around 150 (not shown in Figure 5). Experimentally, the coating solution containing 12 wt.% AF-MWNTs was inhomogeneous due to the formation of polymer agglomerates. Presumably, this inhomogeneity could be caused by the rigidification of polymer chains by the excessive amount of MWNTs [38].

### 3.4. Effect of AF-MWNT Loading on Membrane Performance at a High Feed Pressure

**Figure 6:**
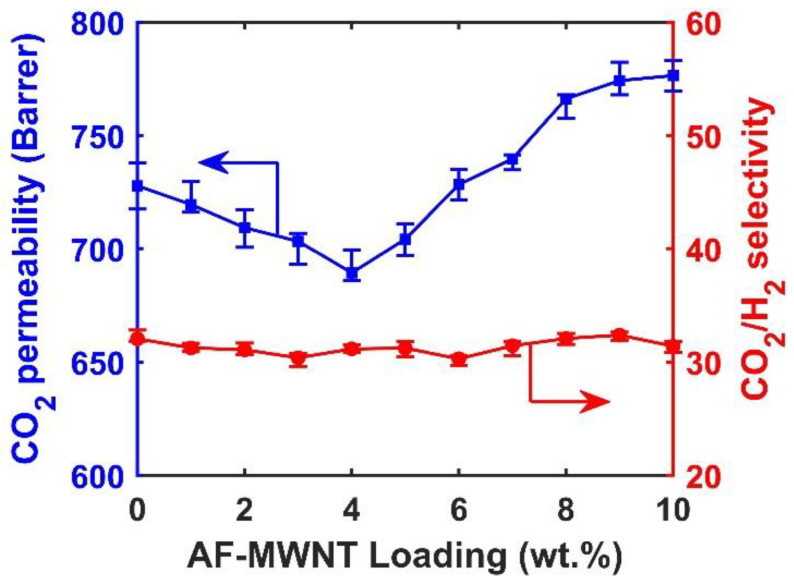
Effects of AF-MWNT loading on CO_2_ permeability and CO_2_/H_2_ selectivity under 1.5-MPa feed pressure: CO_2_ permeability (■) and CO_2_/H_2_ selectivity (●). Error bars represent the minimum and maximum values of three samples.

Figure 6 shows the transport performance of membranes with different AF-MWNT loadings at a feed pressure of 1.5 MPa and 107 °C. There was a reduction of the CO_2_ permeability when the concentration of AF-MWNT increased from 0 to 4 wt.%, dropping from 728 to 689 Barrers. As the concentration of AF-MWNT further increased to 8 wt.%, the CO_2_ permeability significantly increased and reached 766 Barrers. When the concentration of AF-MWNT was increased to 10 wt.%, the CO_2_ permeability increased slightly to 776 Barrers. Within the range of 0–10 wt.% AF-MWNT loadings, the CO_2_/H_2_ selectivity was nearly constant around 31.

Compared to the transport performance of the membrane operated under a lower feed pressure (see Figure 5), both the CO_2_ permeability and CO_2_/H_2_ selectivity were lower, which was due to the carrier saturation phenomenon [11]. As the solution–diffusion transport of CO_2_ is insignificant compared to the facilitated transport, a further increase of the CO_2_ partial pressure does not lead to any increase of CO_2_ flux after the entirety of the carrier has reacted with CO_2_. Eventually, the CO_2_ flux reaches a plateau, resulting in a decreased CO_2_ permeability. Meanwhile, the flux of H_2_ increases with increasing feed pressure, resulting in a decreased CO_2_/H_2_ selectivity.

**Figure 7:**
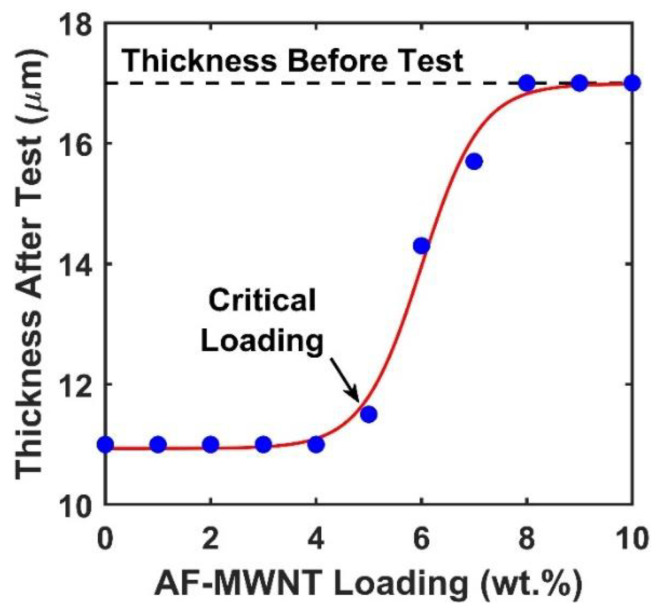
Effect of AF-MWNT loading on the membrane thickness after testing under 1.5-MPa feed pressure for 12 h. The red sigmoidal curve is the best fitted with a log function.

It should be noted that at 1.5-MPa feed pressure, the membrane with 10 wt.% AF-MWNT loading exhibited a higher CO_2_ permeability than that of the membrane without the nanofiller. On the other hand, the incorporation of AT-MWNTs always resulted in a reduced CO_2_ permeability at the 0.2-MPa feed pressure (See Figure 5). In order to understand this difference, the thicknesses of membranes after operated under 1.5-MPa feed pressure for 12 h were measured, and the results are presented in Figure 7. The SEM cross-sectional images were used to measure the thicknesses of the selective layers, which were all at ca. 17 μm before testing. 

As seen from Figure 7, the data approximately followed a sigmoidal curve, indicating that certain critical percolation of the AF-MWNTs was required to trigger the anti-compaction effect. At an AF-MWNT loading in the range of 0–4 wt.%, the membrane thicknesses all reduced to ca. 11 μm because of the severe membrane compaction. When the loading of AF-MWNT increased from 4 wt.% to 8 wt.%, the membrane thicknesses after the tests gradually increased to 17 μm and remained unchanged for an even higher nanofiller loading. This set of experiments suggested the existence of a critical loading of ca. 5 wt.%, below which no anti-compaction effect could be observed. It should be noted that the AF-MWNTs possessed a high aspect ratio of 100–1000. The majority of the AF-MWNTs should be positioned perpendicular to the membrane thickness direction because of the shear-induced alignment during the coating step of membrane fabrication [15]. From the viewpoint of micromechanics, the hydrostatic force is exerted on the thickness direction, and the load transfer occurs on the radial direction, rather than the axial direction, of the nanotubes. In this case, the high tensile strength of the nanotube does not provide any anti-compaction effect; rather, fairly high loading of the nanotube is required to restrict the local polymer deformation [39,40].

Therefore, the transport performances of the membranes with different loadings of AF-MWNTs shown in Figure 6 were a consequence of the trade-off between the dilution effect and the reinforcement effect. For the membranes with a low AF-MWNT loading (0–4 wt.%), the incorporation of AF-MWNTs reduced the AC value, thus, reducing the CO_2_ permeability. When the concentration of AF-MWNT was above 5 wt.%, the reinforcement effect compensated for the adverse influence of the dilution, which resulted in the increasing CO_2_ permeability at a high nanofiller loading.

### 3.5. Comparison with Literature Data

**Figure 8:**
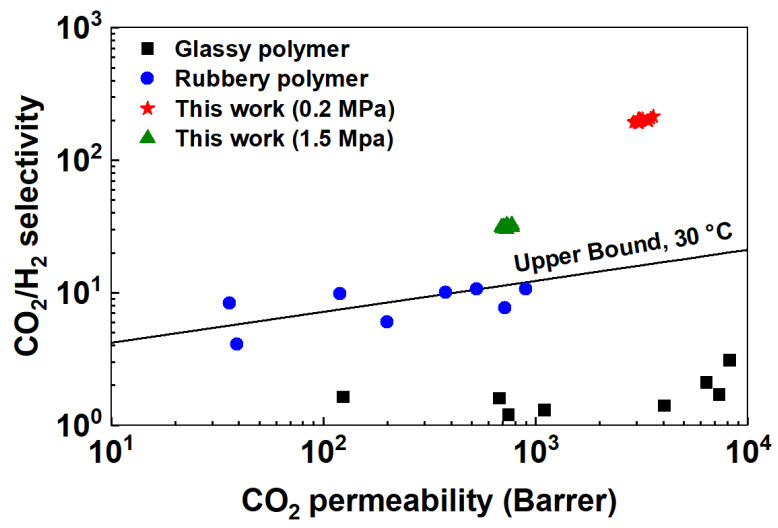
Transport performances of facilitated transport membranes (FTMs) incorporated with AF-MWNTs at 0.2-MPa feed pressure (★) and 1.5-MPa feed pressure (▲) in this work vs. representative glassy polymers (■) and rubbery polymers (●) from the literature [41]. The theoretical upper bound is drawn according to the prediction by Lin et al. for rubbery polymers [42,43].

Figure 8 presents the transport performances of the FTMs incorporated with AF-MWNTs in this work with other polymeric membranes from the literature [41]. The theoretical upper bound in the figure, derived by Lin et al. for rubbery polymers, gives an estimate of the highest selectivity possible for a given permeability based on the solution-diffusion mechanism at 30 °C [42,43]. As shown in the figure, the representative transport performances of the amine-containing membranes, especially the membranes under low feed pressure, were far above the theoretical upper bound at 30 °C. It should be noted that the location of the CO_2_/H_2_ upper bound is temperature-dependent and it moves downwards with increasing temperature [44]. Therefore, the performances of the amine-containing membranes in this work are far above the upper bound at 107 °C. In comparison, the CO_2_/H_2_ selectivities of glassy polymers were low, i.e., 1–2, due to their size sieving feature, which favors the diffusion of H_2_ [41]. Meanwhile, the rubbery polymers, which possessed functional groups that are affinitive to CO_2_ solubility, were sitting around the upper bound [41]. Therefore, compared to these materials, the membranes synthesized in this work exhibited superior performances due to the facilitated transport mechanism.

### 3.6. Effect of AF-MWNT Loading on Membrane Stability

**Figure 9:**
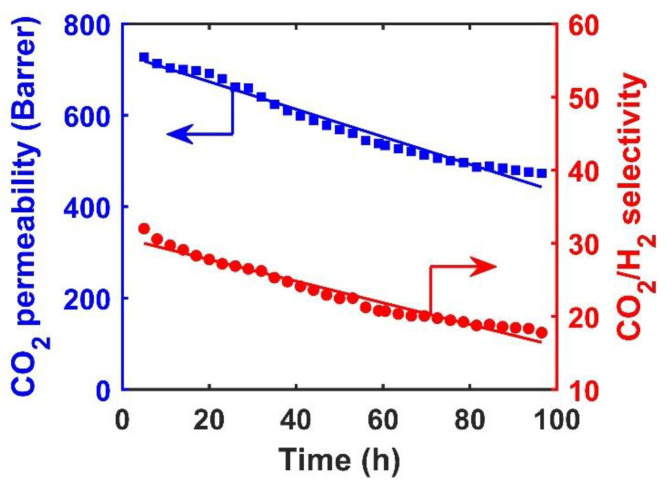
Transport performance of membrane without AF-MWNTs at 107 °C and 1.5-MPa feed pressure over a course of 100 h: CO_2_ permeability (■) and CO_2_/H_2_ selectivity (●).

The reinforcement effect of the AF-MWNTs was also studied in terms of the membrane stability at an elevated feed pressure. Figure 9 depicts the transport performance of the membrane without nanofillers under 1.5-MPa feed pressure and 107 °C over the course of 100 h. As seen, both the CO_2_ permeability and CO_2_/H_2_ selectivity showed gradual reduction during the 100-h test by approximately 35% and 40%, respectively. In addition, no sign of stabilization was observed by the end of the test. 

**Figure 10:**
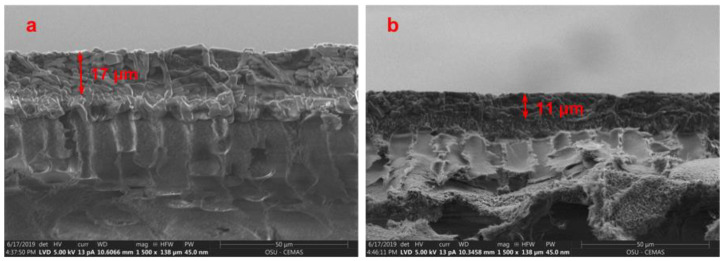
Cross-sectional SEM images of membrane without AF-MWNTs (**a**) before and (**b**) after the 100-h test at 1.5 MPa.

Figure 10 shows the cross-sectional morphologies of the membrane before and after the 100-h test. There was an obvious reduction in the thickness of the selective layer from 17 to 11 μm. The reduction was due to the compaction effect, which played a significant role in unstable membrane performance. Without the nanofillers, the compressed selective layer resulted in the reduction of free volume in the membrane. Therefore, the diffusivities of mobile carriers and gas molecules were reduced, which resulted in a slower CO_2_ permeation across the membrane [45]. 

**Figure 11:**
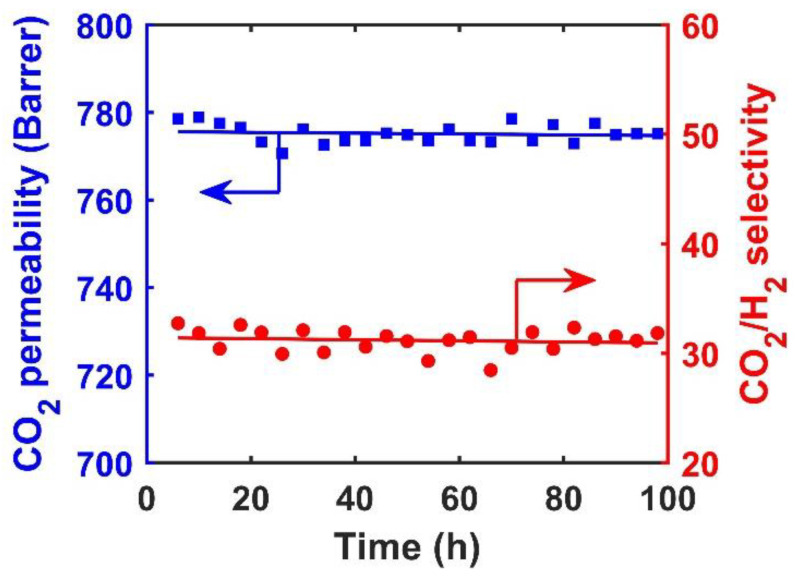
Stability of membrane with 10 wt.% AF-MWNTs at 107 °C and 1.5 MPa: CO_2_ permeability (■) and CO_2_/H_2_ selectivity (●).

Figure 11 depicts the stability of the membrane containing 10 wt.% AF-MWNTs at 1.5 MPa and 107 °C for 100 h. During this period, the performance of the membrane was stable, and there was no sign of degradation for the gas permeation performance with the maintained CO_2_ permeability of 776 Barrers and CO_2_/H_2_ selectivity of 31. 

**Figure 12:**
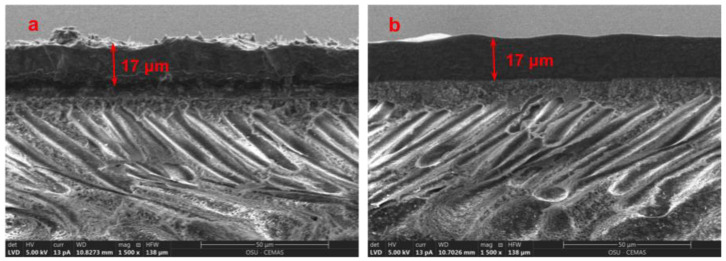
Cross-sectional SEM images of membrane with 10 wt.% AF-MWNTs (**a**) before and (**b**) after the 100-h stability test at 1.5 MPa.

Figure 12 shows the cross-sectional SEM images of this membrane before and after the 100-h test. The thickness of the selective layer, i.e., 17 μm, was maintained throughout the 100-h test. Interestingly, after testing, the selective layer becomes smoother than the membrane prior to the test. Presumably, the rough surface might be caused by the protrusion of MWNTs during the drying step of membrane preparation [14]. Apparently, the rough surface was smoothened out with the leveling of MWNTs under the high-pressure humid condition during the stability testing. Compared to the membrane with no nanofiller, the membrane with 10 wt.% AF-MWNTs exhibited remarkable improvement not only for CO_2_/H_2_ separation performance but also for membrane stability.

## 4. Conclusions

In this work, different loadings of AF-MWNT were incorporated in the amine-containing membrane with crosslinked PVA–POS as the polymer matrix for CO_2_/H_2_ separation at a feed pressure up to 1.5 MPa and a relatively high temperature of 107 °C. At 0.2-MPa feed pressure, the membrane incorporated with 10 wt.% AF-MWNTs showed a CO_2_ permeability of 3196 Barrers and a CO_2_/H_2_ selectivity of 205, which were lower than those of the membrane without the nanofiller due to the dilution effect by the AF-MWNTs. At 1.5-MPa feed pressure, the same membrane exhibited the best transport performance, showing a CO_2_ permeability of 776 Barrers and a CO_2_/H_2_ selectivity of 31, owing to the reinforcement effect of the AF-MWNTs. Furthermore, the incorporation of 10 wt.% AF-MWNTs led to a significant improvement on the membrane stability under 1.5-MPa feed pressure, which suggested that the incorporation of AF-MWNTs improved the resistance to membrane compaction upon a high feed pressure. In addition, the representative transport performances of all membranes under 0.2-MPa and 1.5-MPa feed pressures well surpassed the theoretical upper bound. Therefore, this work is considered as one of the crucial steps to enable the application of facilitated transport membranes to high-pressure gas processing, such as syngas purification. 

## Figures and Tables

**Table 1:** Operating conditions for gas transport performance measurements.

T (°C)	Pressure (MPa)		Dry Gas Flow Rate (cm^3^/min)		Relative Humidity (%)	
	Feed	Sweep	Feed	Sweep	Feed	Sweep
107	0.2	0.1	100	30	59.2	47.4
107	1.5	0.1	100	30	100	72.5

**Table 2:** Calculated amine concentration (AC) values for amine carriers.

Carriers	AC Value (mmol/g)
PVAm	23.3
AIBA-K	14.4
APTES	4.5

## References

[1] Roh H.-S., Jun K.-W., Dong W.-S., Chang J.-S., Park S.-E., Joe Y.-I. (2002). Highly active and stable Ni/Ce-ZrO_2_ catalyst for H_2_ production from methane. J. Mol. Catal. A Chem..

[2] Merkel T.C., Zhou M., Baker R.W. (2012). Carbon dioxide capture with membranes at an IGCC power plant. J. Membr. Sci..

[3] Momirlan M., Veziroglu T.N. (2005). The properties of hydrogen as fuel tomorrow in sustainable energy system for a cleaner planet. Int. J. Hydrogen Energy.

[4] Ramasubramanian K., Zhao Y., Ho W.S.W. (2013). CO_2_ capture and H_2_ purification: Prospects for CO_2_-selective membrane processes. AlChE J..

[5] Dijkstra J., Jansen D. (2004). Novel concepts for CO_2_ capture. Energy.

[6] Ho W.S.W., Sirkar K. (2012). Membrane Handbook.

[7] Ho W.S.W., Dalrymple D. (1994). Facilitated transport of olefins in Ag^+^-containing polymer membranes. J. Membr. Sci..

[8] Zhao Y., Ho W.S.W. (2012). CO_2_-selective membranes containing sterically hindered amines for CO_2_/H_2_ separation. Ind. Eng. Chem. Res..

[9] Quinn R., Laciak D., Pez G. (1997). Polyelectrolyte-salt blend membranes for acid gas separations. J. Membr. Sci..

[10] Okada O., Teramoto M., Yegani R., Matsuyama H., Shimada K., Morimoto K. (2012). CO2-Facilitated Transport Membrane and Method for Producing the Same. U.S. Patent.

[11] Zou J., Ho W.S.W. (2006). CO_2_-selective polymeric membranes containing amines in crosslinked poly(vinyl alcohol). J. Membr. Sci..

[12] Deng L., Kim T.-J., Hägg M.-B. (2009). Facilitated transport of CO_2_ in novel PVAm/PVA blend membrane. J. Membr. Sci..

[13] Dimilia R.A., Reed J.S. (1983). Dependence of compaction on the glass transition temperature of the binder plase. Am. Ceram. Soc. Bull..

[14] Zhao Y., Jung B.T., Ansaloni L., Ho W.S.W. (2014). Multiwalled carbon nanotube mixed matrix membranes containing amines for high pressure CO_2_/H_2_ separation. J. Membr. Sci..

[15] Han Y., Wu D., Ho W.S.W. (2018). Nanotube-reinforced facilitated transport membrane for CO_2_/N_2_ separation with vacuum operation. J. Membr. Sci..

[16] Deng L., Hägg M.-B. (2014). Carbon nanotube reinforced PVAm/PVA blend FSC nanocomposite membrane for CO_2_/CH_4_ separation. Int. J. Greenh. Gas Con..

[17] Ansaloni L., Zhao Y., Jung B.T., Ramasubramanian K., Baschetti M.G., Ho W.S.W. (2015). Facilitated transport membranes containing amino-functionalized multi-walled carbon nanotubes for high-pressure CO_2_ separations. J. Membr. Sci..

[18] Van Damme M., Sap W., Van Aert H. (2004). Processless Lithographic Printing Plate. U.S. Patent.

[19] Morgan M., Fielding L., Armes S. (2013). Synthesis and characterisation of sterically stabilised polypyrrole particles using a chemically reactive poly(vinyl amine)-based stabiliser. Colloid. Polym. Sci..

[20] De Lannoy C.-F., Soyer E., Wiesner M.R. (2013). Optimizing carbon nanotube-reinforced polysulfone ultrafiltration membranes through carboxylic acid functionalization. J. Membr. Sci..

[21] Shanmugharaj A., Bae J., Lee K.Y., Noh W.H., Lee S.H., Ryu S.H. (2007). Physical and chemical characteristics of multiwalled carbon nanotubes functionalized with aminosilane and its influence on the properties of natural rubber composites. Compos. Sci. Technol..

[22] Xing R., Ho W.S.W. (2011). Crosslinked polyvinylalcohol–polysiloxane/fumed silica mixed matrix membranes containing amines for CO_2_/H_2_ separation. J. Membr. Sci..

[23] Chen Y., Ho W.S.W. (2016). High-molecular-weight polyvinylamine/piperazine glycinate membranes for CO_2_ capture from flue gas. J. Membr. Sci..

[24] Mawhinney D.B., Naumenko V., Kuznetsova A., Yates J.T., Liu J., Smalley R. (2000). Infrared spectral evidence for the etching of carbon nanotubes: Ozone oxidation at 298 K. J. Am. Chem. Soc..

[25] Shaffer M.S., Fan X., Windle A. (1998). Dispersion and packing of carbon nanotubes. Carbon.

[26] Lee G.-W., Kim J., Yoon J., Bae J.-S., Shin B.C., Kim I.S., Oh W., Ree M. (2008). Structural characterization of carboxylated multi-walled carbon nanotubes. Thin Solid Films.

[27] Bai H., Ho W.S.W. (2011). Carbon dioxide-selective membranes for high-pressure synthesis gas purification. Ind. Eng. Chem. Res..

[28] Kathi J., Rhee K.-Y., Lee J.H. (2009). Effect of chemical functionalization of multi-walled carbon nanotubes with 3-aminopropyltriethoxysilane on mechanical and morphological properties of epoxy nanocomposites. Compos. Part A Appl. Sci. Manuf..

[29] Socrates G. (2001). Infrared and Raman Characteristic Group Frequencies.

[30] Du J., Wang J., Su S., Wilkie C.A. (2004). Additional XPS studies on the degradation of poly (methyl methacrylate) and polystyrene nanocomposites. Polym. Degrad. Stab..

[31] Graf N., Yegen E., Gross T., Lippitz A., Weigel W., Krakert S., Terfort A., Unger W.E. (2009). XPS and NEXAFS studies of aliphatic and aromatic amine species on functionalized surfaces. Surf. Sci..

[32] Beard B.C. (1990). Cellulose nitrate as a binding energy reference in N (1s) XPS studies of nitrogen-containing organic molecules. Appl. Surf. Sci..

[33] Dietrich P.M., Streeck C., Glamsch S., Ehlert C., Lippitz A., Nutsch A., Kulak N., Beckhoff B., Unger W. (2015). Quantification of silane molecules on oxidized silicon: Are there options for a traceable and absolute determination?. Anal. Chem..

[34] Jakša G., Štefane B., Kovač J. (2013). XPS and AFM characterization of aminosilanes with different numbers of bonding sites on a silicon wafer. Surf. Interface Anal..

[35] Martin H.J., Schulz K.H., Bumgardner J.D., Walters K.B. (2007). XPS study on the use of 3-aminopropyltriethoxysilane to bond chitosan to a titanium surface. Langmuir.

[36] Peigney A., Laurent C., Flahaut E., Bacsa R., Rousset A. (2001). Specific surface area of carbon nanotubes and bundles of carbon nanotubes. Carbon.

[37] Ge L., Zhu Z., Rudolph V. (2011). Enhanced gas permeability by fabricating functionalized multi-walled carbon nanotubes and polyethersulfone nanocomposite membrane. Sep. Purif. Technol..

[38] Skoulidas A.I., Sholl D.S., Johnson J.K. (2006). Adsorption and diffusion of carbon dioxide and nitrogen through single-walled carbon nanotube membranes. J. Chem. Phys..

[39] Kumar P.P., Rao V.S., Chandra S.S. (2018). Deflection behavior of carbon nanotube reinforced polymer composite beams using first order shear deformation theory. Mater. Today.

[40] MacDonald R.A., Laurenzi B.F., Viswanathan G., Ajayan P.M., Stegemann J.P. (2005). Collagen–carbon nanotube composite materials as scaffolds in tissue engineering. J. Biomed. Mater. Res..

[41] Han Y., Ho W.S.W. (2018). Recent advances in polymeric membranes for CO_2_ capture. Chin. J. Chem. Eng..

[42] Freeman B.D. (1999). Basis of permeability/selectivity tradeoff relations in polymeric gas separation membranes. Macromolecules.

[43] Lin H., Van Wagner E., Freeman B.D., Toy L.G., Gupta R.P. (2006). Plasticization-enhanced hydrogen purification using polymeric membranes. Science.

[44] Rowe B.W., Robeson L.M., Freeman B.D., Paul D.R. (2010). Influence of temperature on the upper bound: Theoretical considerations and comparison with experimental results. J. Membr. Sci..

[45] Härtel G., Püschel T. (1999). Permselectivity of a PA6 membrane for the separation of a compressed CO_2_/H_2_ gas mixture at elevated pressures. J. Membr. Sci..

